# Approaching the activity limit of CoSe_2_ for oxygen evolution via Fe doping and Co vacancy

**DOI:** 10.1038/s41467-020-15498-0

**Published:** 2020-04-03

**Authors:** Yuhai Dou, Chun-Ting He, Lei Zhang, Huajie Yin, Mohammad Al-Mamun, Jianmin Ma, Huijun Zhao

**Affiliations:** 1 Centre for Clean Environment and Energy, Gold Coast Campus, Griffith University, Gold Coast, QLD 4222 Australia; 2 Key Laboratory of Functional Small Organic Molecule, Ministry of Education, College of Chemistry and Chemical Engineering, Jiangxi Normal University, Nanchang, Jiangxi 330022 China; 3 School of Physics and Electronics, Hunan University, Changsha, Hunan 410082 China; 4 Centre for Environmental and Energy Nanomaterials, CAS Centre for Excellence in Nanoscience, Institute of Solid State Physics, Chinese Academy of Sciences, Hefei, Anhui 230031 China

**Keywords:** Electrocatalysis, Renewable energy, Electrocatalysis, Nanoscale materials

## Abstract

Electronic structure engineering lies at the heart of efficient catalyst design. Most previous studies, however, utilize only one technique to modulate the electronic structure, and therefore optimal electronic states are hard to be achieved. In this work, we incorporate both Fe dopants and Co vacancies into atomically thin CoSe_2_ nanobelts for /coxygen evolution catalysis, and the resulted CoSe_2_-D_Fe_–V_Co_ exhibits much higher catalytic activity than other defect-activated CoSe_2_ and previously reported FeCo compounds. Deep characterizations and theoretical calculations identify the most active center of Co_2_ site that is adjacent to the V_Co_-nearest surface Fe site. Fe doping and Co vacancy synergistically tune the electronic states of Co_2_ to a near-optimal value, resulting in greatly decreased binding energy of OH* (ΔE_OH_) without changing ΔE_O_, and consequently lowering the catalytic overpotential. The proper combination of multiple defect structures is promising to unlock the catalytic power of different catalysts for various electrochemical reactions.

## Abstract (web summary)

While electronic-structure engineering lies at the heart of catalyst design, most previous studies utilize only one technique to tune the electronic states. Here, authors demonstrate that Fe doping and Co vacancy work synergistically to approach the activity limit of CoSe_2_ for oxygen evolution reaction.

## Introduction

Design of highly efficient water splitting electrocatalysts for clean hydrogen production is essential for the sustainable development of modern society^1,2^. Electronic structure engineering via incorporating dopants, vacancies, strains, heterostructures, etc. lies at the heart of efficient catalyst design, as it effectively tunes the binding energies of reaction intermediates^3,4^. Most previous studies, however, utilize only one technique to manipulate the electronic structure, and thus the catalytic performance is barely satisfactory^5,6^. For instance, CoSe_2_ has moderate catalytic activity toward oxygen evolution reaction (OER), and the doping of Fe has been demonstrated to be an effective approach to improving the performance^7,8^. However, whether the activity could be further enhanced in a synergetic manner by incorporating other defects, such as anion and cation vacancies, has not been reported. We believe that the proper combination of two or more defect structures is essential to achieve near optimal electronic states and ideal intermediate binding energies, which holds the key for the construction of highly efficient water splitting electrocatalysts.

In this work, we seek to fully excavate the catalytic potential of atomically thin CoSe_2_ nanobelts for OER by incorporating Fe dopants and Co/Se vacancies. Through both experiments and theoretical calculations, we find that the best catalyst is CoSe_2_–D_Fe_–V_Co_ and the most active center is the Co_2_ site adjacent to the V_Co_-nearest surface Fe site. Fe doping and Co vacancy work synergistically to optimize the electronic states of Co_2_, and therefore the binding energy of OH* is dramatically decreased and high catalytic activity is achieved. By contrast, Se-derived O vacancy has an obvious impact on the binding energy of O* at Co_2_ site, which results in relatively high overpotential and low catalytic activity.

## Results

### Synthesis and structural characterization

**Fig. 1:**
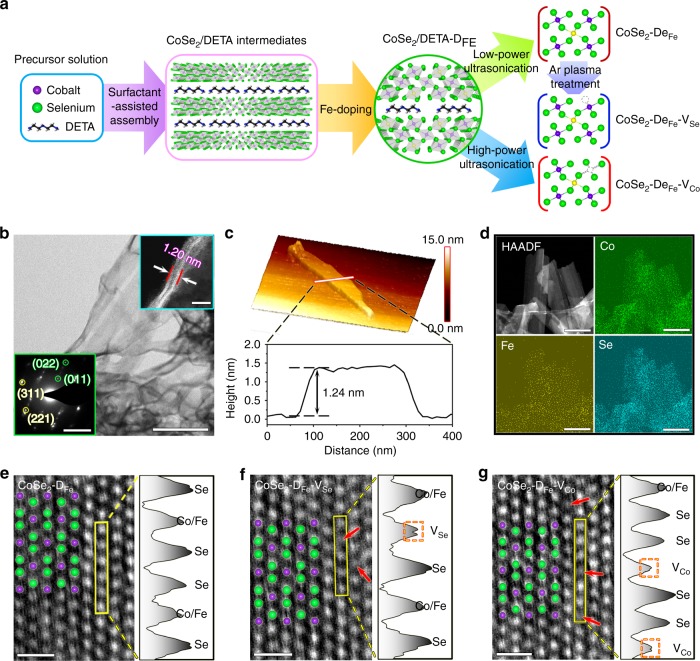
Synthesis and structural characterization of CoSe_2_–D_Fe_, CoSe_2_–D_Fe_–V_Se_, and CoSe_2_–D_Fe_–V_Co_. **a** Schematic illustration of the synthetic methods for different catalysts. **b** Scanning transmission electron microscopy (STEM) image of CoSe_2_–D_Fe_–V_Co_ nanobelts with selected area electron diffraction pattern (left inset) indicating the mixed cubic and orthorhombic phases and cross-sectional view (right inset) showing the atomic thickness. Scale bar, 200 nm, 5 1/nm (left inset) and 2 nm (right inset). **c** Atomic force microscopy image of CoSe_2_–D_Fe_–V_Co_ and height profile along the white line in the image. **d** Elemental maps of Co, Fe, and Se in CoSe_2_–D_Fe_–V_Co_. Scale bar, 300 nm. **e**–**g** High-resolution high-angle annular dark-field STEM (HAADF-STEM) images of different catalysts and the intensity profiles along the selected rectangular regions suggest the missed surface Se and Co atoms in CoSe_2_–D_Fe_–V_Se_ and CoSe_2_–D_Fe_–V_Co_, respectively. Scale bar, 0.5 nm.

Figure 1a shows the synthetic strategies of different electrocatalysts. Firstly, CoSe_2_ nuclei were assembled with diethylenetriamine (DETA) under hydrothermal reaction, leading to the formation of CoSe_2_/DETA lamellar intermediates^9^. Fe ions were then incorporated into CoSe_2_/DETA via a wet-impregnation method involving chemical adsorption and cation exchange^10^. After that, atomically thin CoSe_2_–D_Fe_ nanobelts were obtained by liquid-phase exfoliation using low-power ultrasonication. Se vacancies were created on the surface of exfoliated CoSe_2_–D_Fe_ nanobelts through Ar plasma treatment^11^, and Co vacancies were extracted by DETA molecules during the exfoliation of CoSe_2_/DETA under high ultrasonic power^12^. Figure 1b shows the scanning transmission electron microscopy (STEM) image of the CoSe_2_–D_Fe_–V_Co_ nanobelts. The selected area electron diffraction (SAED) pattern (left inset) reveals the coexistence of cubic (yellow) and orthorhombic (green) phases of CoSe_2_ matrix, which is also identified by the X-ray diffraction (XRD) patterns (Supplementary Fig. 1)^13^. The cross-sectional view of the nanobelts (right inset) suggests an atomic thickness of ~1.20 nm, which is further confirmed by the atomic force microscopy analysis (Fig. 1c). Energy dispersive X-ray spectroscopy (EDS) mapping shows that Co, Se, and Fe dopants are homogeneous distributed throughout the nanobelts (Fig. 1d). The doping ratio of Fe to total cation content is about 18.3% according to the inductively coupled plasma atomic emission spectroscopy (ICP-AES) results (Supplementary Table 1). A certain amount of O was also detected by EDS analysis (Supplementary Fig. 2), attributing to the deviation of Se/Co ratio from the stoichiometry of CoSe_2_ that results from the low solubility of Na_2_SeO_3_ in H_2_O-DETA mixed solvent^13,14^. The presence of oxides was also evidenced by Raman spectroscopy (Supplementary Fig. 3), where the peak at 164 cm^−1^ corresponds to the stretching mode of Se–Se in CoSe_2_^12^, and the peaks at 190, 515, 612, 472, and 676 cm^−1^ are assigned to the F_2g_(1), F_2g_(2), F_2g_(3), E_g_, and A_1g_ modes of CoO_*x*_, respectively^15^. The created Se and Co vacancies are readily visible via high-angle annular dark-field STEM (HAADF-STEM) imaging (Fig.1e–g). In addition, the intensity profiles along the selected rectangular regions also suggest the missed surface Se and Co atoms in CoSe_2_–D_Fe_–V_Se_ and CoSe_2_–D_Fe_–V_Co_, respectively.

### OER catalytic activity evaluation

**Fig. 2:**
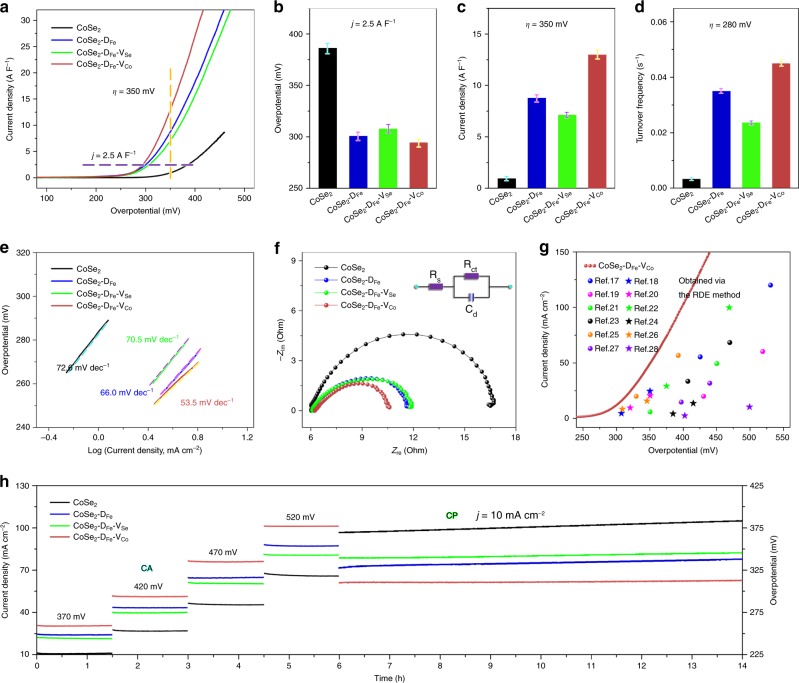
Catalytic performance for the oxygen evolution reaction. **a** Linear sweep voltammetry curves normalized by electrochemical double-layer capacitance. **b** Overpotentials (*η*) required to reach a current density (*j*) of 2.5 A F^−1^. **c** Current densities at *η* = 350 mV. **d** Turnover frequencies calculated at *η* = 280 mV. **e** Tafel plots derived from the polarization curves in low overpotential regions. **f** Nyquist plots at *η* = 370 mV with inset showing the equivalent circuit model. **g** Comparison of the catalytic performance between CoSe_2_–D_Fe_–V_Co_ and previously reported CoSe_2_ and FeCo compounds evaluated via the rotating disk electrode (RDE) method. **h** Durability evaluation via chronoamperometry test at stepwise *η* of 370, 420, 470, and 520 mV for 6 h, and chronopotentiometry test at *j* = 10 mA cm^−2^ for 8 h. The error bars in **b**–**d** denote standard deviation of five technical replicates.

To understand how Fe dopants and Co/Se vacancies affect the OER catalytic performance, the prepared catalysts were subjected to systematic electrochemical evaluation via the rotating disk electrode (RDE) method in purified 1 M NaOH (Fig. 2). Cyclic voltammetry (CV) was first conducted to activate the electrodes (Supplementary Fig. 4), and large current densities were recorded during the first few cycles, indicating the instability of CoSe_2_ in alkaline solution at anodic potentials^14^. EDS analysis shows that Se signal can hardly be detected after CV test, and SAED pattern indicates the formation of CoOOH phase, which serves as the real host under OER conditions (Supplementary Fig. 5). The thickness of the nanobelts increases from 1.24 to 1.35 nm after CV activation (Supplementary Fig. 6), which is resulted from the significantly increased surface roughness due to CV-induced structural reconstruction.

The catalytic activities were then assessed by linear sweep voltammetry (LSV, Fig. 2a). The polarization curves were corrected with 95% iR-compensation and then normalized by the electrochemical double-layer capacitance. As shown in Fig. 2b, c, to reach a current density (*j*) of 2.5 A F^−1^, the required overpotentials (*η*) for CoSe_2_, CoSe_2_–D_Fe_, CoSe_2_–D_Fe_–V_Se_, and CoSe_2_–D_Fe_–V_Co_ are 385.7, 300.4, 307.3, and 294.2 mV, respectively, and at *η* = 350 mV, the recorded *j* are 0.92, 8.73, 7.06, and 12.95 A F^−1^, respectively. Therefore, Fe doping, in accordance with previous reports, enhances the OER catalytic performance of CoSe_2_, and further incorporation of Se and Co vacancies leads to slightly decreased and dramatically increased catalytic activity, respectively. It is important to note that the current density achieved by CoSe_2_–D_Fe_–V_Co_ at *η* = 350 mV is 30.2% higher than the sum of the current densities of CoSe_2_–D_Fe_ and CoSe_2_–V_Co_ (Supplementary Fig. 7), indicating a synergistic effect between Fe dopants and Co vacancies. This synergistic effect was further confirmed by changing the sequence of Fe doping and Co vacancy. As shown in Supplementary Fig. 8, the CoSe_2_–V_Co_–D_Fe_ exhibits higher overpotential and lower current density than CoSe_2_–D_Fe_–V_Co_, probably due to that the afterward doping fills the vacancy sites and weakens the role of Co vacancies^16^.

The high catalytic activity of CoSe_2_–D_Fe_–V_Co_ was further evidenced by analyzing the turnover frequency (TOF, assuming all cations to be catalytically active) and Tafel slope (derived from the polarization curve in low overpotential region). As shown in Fig. 2d, the apparent TOF of CoSe_2_–D_Fe_–V_Co_ at *η* = 280 mV reaches 0.045 s^−1^, exceeding 0.003, 0.035, and 0.024 s^−1^ for CoSe_2_, CoSe_2_–D_Fe_, and CoSe_2_–D_Fe_–V_Se_, respectively, indicative of the high intrinsic catalytic activity. In addition, CoSe_2_–D_Fe_–V_Co_ exhibits a small Tafel slope of 53.5 mV dec^−1^, much lower than 72.6, 66.0, and 70.5 mV dec^−1^ for CoSe_2_, CoSe_2_–D_Fe_ and CoSe_2_–D_Fe_–V_Se_, respectively (Fig. 2e), suggesting the remarkably enhanced reaction kinetics^6^. The Nyquist plots at *η* = 370 mV display typical semicircles for different catalysts (Fig. 2f), corresponding to the charge transfer resistances (*R*_ct_). Using a relevant equivalent circuit (inset), the fitted results indicate the smallest *R*_ct_ (4.2 Ω) of CoSe_2_–D_Fe_–V_Co_ compared with those of CoSe_2_ (10.5 Ω), CoSe_2_–D_Fe_ (5.3 Ω) and CoSe_2_–D_Fe_–V_Se_ (5.4 Ω), indicating the efficient electron transfer and the fast ion diffusion^8^. As a result, the incorporation of Fe dopants and Co vacancies creates highly active catalytic sites with excellent mass transport properties for OER catalysis.

It is also noteworthy that CoSe_2_–D_Fe_–V_Co_ outperforms commercial IrO_2_ and RuO_2_ electrodes (Supplementary Fig. 9), and previously reported CoSe_2_ and FeCo compounds evaluated via the RDE method (Fig. 2g)^17–28^. The outstanding performance is firstly attributable to the atomic thickness of the nanobelts, which provides abundant surface active sites for the catalytic reaction (Supplementary Fig. 10). In addition, the proper doping level of Fe optimizes the electrode composition and improves the catalytic performance (Supplementary Fig. 11). More importantly, Fe doping and Co vacancies work synergistically to create highly active centers and dramatically enhance the catalytic activity.

The catalytic stability was further assessed via chronoamperometry (CA) for 6 h and chronopotentiometry (CP) for 8 h. As shown in Fig. 2h, CA test shows that the current density increases with increasing overpotential. More specifically, CoSe_2_–D_Fe_–V_Co_ delivers average current densities of 30.5, 51.6, 76.3, 101.6 mA cm^−2^ at *η* = 370, 420, 470, 520 mV, respectively, exceeding those of CoSe_2_ (10.8, 26.9, 45.5, 66.3 mA cm^−2^), CoSe_2_–D_Fe_ (24.6, 43.5, 64.5, 87.0 mA cm^−2^), and CoSe_2_–D_Fe_–V_Se_ (21.6, 39.8, 60.5, 80.5 mA cm^−2^). CP test at *j* = 10 mA cm^−2^ shows that the overpotential of CoSe_2_ suffers an obvious increase of 13.6 mV, while those of CoSe_2_–D_Fe_, CoSe_2_–D_Fe_–V_Se_, and CoSe_2_–D_Fe_–V_Co_ see slight rises of 8.6, 7.0, and 3.7 mV, respectively, which should be attributed to the enhanced electrical conductivity caused by Fe doping^29^. Generally, the trend in the activity of different catalysts does not change after long-term durability test.

### Understanding defect structures before and after catalysis

**Fig. 3:**
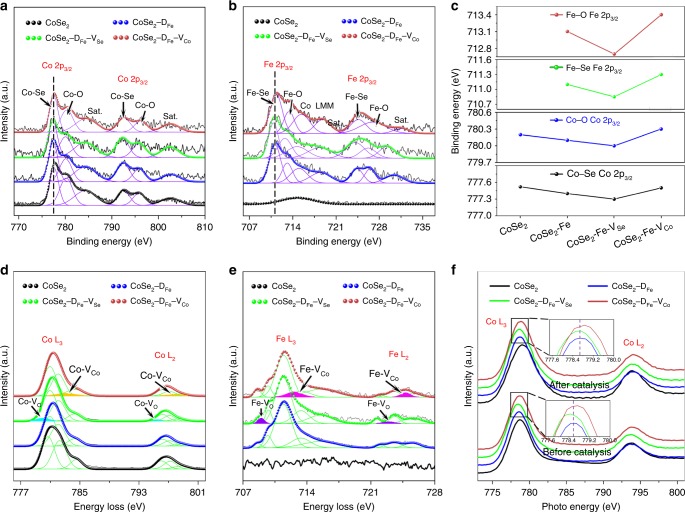
Defect structure identification before and after catalysis. **a**, **b** Co 2p and Fe 2p X-ray photoelectron spectroscopy spectra of primary catalysts. **c** Binding energy shifts of Co–Se, Co–O, Fe–Se, and Fe–O peaks due to doping and vacancies. **d**, **e** Co and Fe L-edge electron energy loss spectra after chronoamperometry test at 370 mV for 1.5 h showing the existence of extra peaks in CoSe_2_–D_Fe_–V_Se_ and CoSe_2_–D_Fe_–V_Co_. **f** Co L-edge X-ray absorption near-edge structure spectra before and after catalysis showing the energy shifts caused by doping and vacancies.

Prior to the mechanism investigation, it is essential to study the stability of dopants and vacancies and probe the evolution of electronic structures during catalysis as they are directly relevant to the catalytic activity^30^. X-ray photoelectron spectroscopy (XPS) was first performed to understand the primary defects before catalysis. As shown in Fig. 3a, b and Supplementary Fig. 12, Co, Fe, and Se signals are clearly detected, indicating the successful doping of Fe into CoSe_2_ matrix, and the Co/Fe 2p spectra show the existence of Co/Fe–O bonds due to oxide impurities. The Co/Fe 2p_3/2_ peaks of Co/Fe–Se and Co/Fe–O in CoSe_2_–D_Fe_–V_Se_ exhibit negative shifts to lower binding energies, and those in CoSe_2_–D_Fe_–V_Co_ show positive shifts to higher binding energies, indicating the reduction and oxidation states of metal sites caused by Se/O and Co vacancies, respectively (Fig. 3c)^31^. The defect structures after CA test at *η* = 370 mV for 1.5 h were then investigated via electron energy loss spectroscopy (EELS). As shown in Fig. 3d, e, both Co and Fe signals can be detected after catalysis, and no significant changes in their atom ratios were found due to fully purified electrolyte (Supplementary Table 1). For better analysis, the L_2,3_-edge spectra of Co and Fe were fitted by multiple Gaussian functions, which represent different oxidation states and coordination environments^32^. Compared with CoSe_2_−D_Fe_, there are extra Gaussian peaks centered at 780.0 and 795.4 eV (cyan) in Co L_2,3_-edge spectra, and 709.0 and 723.0 eV (purple) in Fe L_2,3_-edge spectra of CoSe_2_–D_Fe_–V_Se_, which most likely result from the Se-derived O vacancies. In comparison, extra peaks located at 783.0 and 797.9 eV (orange) in Co L_2,3_-edge spectra, and 712.6 and 724.8 eV (magenta) in Fe L_2,3_-edge spectra of CoSe_2_–D_Fe_–V_Co_, which should be ascribed to the local chemical environment of Co vacancies. Therefore, the surface reconstruction induced by OER catalysis has little impact on the defect structures. To provide further evidence, X-ray absorption near-edge structure (XANES) spectra at the L-edges of Co and Fe before and after catalysis were conducted (Fig. 3f and Supplementary Fig. 13). Obviously, the L_3_-edge centroids of Co before catalysis shift by 0.10 and 0.12 eV to lower and higher energy positions after the incorporation of Se/O and Co vacancies, respectively^33,34^. After catalysis, slight broadenings of the white line peaks were observed due to OER-induced complex coordination environment^35^. Despite the electronic-structure changes, the Co L_3_-edge centroids of CoSe_2_–D_Fe_–V_Se_ and CoSe_2_–D_Fe_–V_Co_ still exhibit 0.06 and 0.11 eV shifts to lower and higher energy positions, respectively. The same trends of energy shift could be obtained from the L_2,3_-edge XANES spectra of Fe (Supplementary Fig. 13). As a result, both dopants and vacancies are well preserved during OER catalysis, and they consequently determine the catalytic performance of different catalysts.

## Discussion

**Fig. 4:**
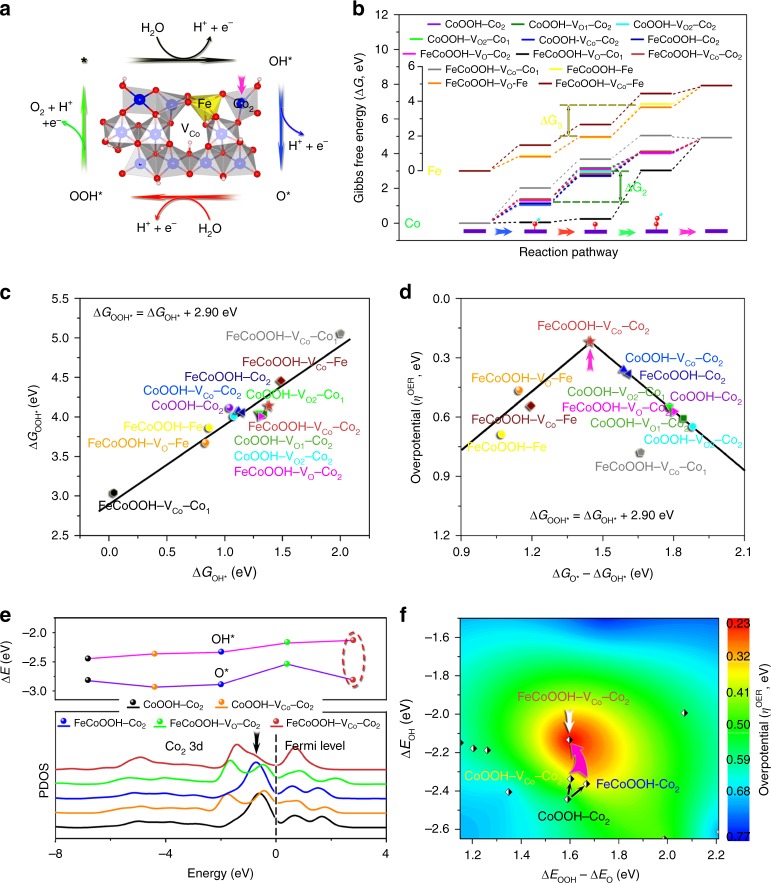
Hubbard-corrected density functional theory calculations of catalytic activities of different surface metal sites. **a** Crystal structure of FeCoOOH–V_Co_ and oxygen evolution reaction reaction pathway on (01-12) facet. **b** Gibbs free energy profiles along the reaction pathway. **c** Scaling relation between Δ*G*_OOH*_ and ΔG_OH*_. **d** Volcano plot of overpotential as a function of Δ*G*_O*_ − Δ*G*_OH*_ based on the scaling relation. **e** Calculated partial density of states (PDOS) of Co_2_ sites and binding energies of OH* and O*. **f** Contour plot of theoretical overpotential as a function of Δ*E*_OOH_ − Δ*E*_O_ and Δ*E*_OH_, indicating the near-optimal intermediate binding energies achieved by Fe doping and Co vacancy.

To provide clear insight into the effects of dopants and vacancies on the catalytic activities, we performed Hubbard-corrected density functional theory (DFT + U) calculations. The crystal structure of CoOOH was utilized to build up the periodical surface models as it serves as the real host under OER conditions (Supplementary Figs. 4 and 5). The high-index (01-12) facet was selected as the surface termination due to the proved good coincidence between theoretical and experimental results^36^, and all potential active sites on (01-12) facets of models with different atomistic arrangements of dopants and vacancies were considered to gain insight into the catalytic mechanism (Supplementary Fig. 14). Figure 4a shows the FeCoOOH–V_Co_ structure and the OER pathway, which involves four proton coupled electron transfer steps and three intermediates of OH*, O*, and OOH*^37^. The corresponding Gibbs free energies (Δ*G*_*i*_, *i* = OH*, O*, and OOH*) were calculated and provided in Fig. 4b. As can be seen, the catalytic activities of Co sites are restricted by the transformation of OH* to O*, due to the relatively large difference between Δ*G*_O*_ and Δ*G*_OH*_ (namely Δ*G*_2_). In comparison, the potential-limiting step of Fe sites is assigned to the transformation of O* to OOH*, owing to the relatively large difference between Δ*G*_OOH*_ and Δ*G*_O*_ (i.e., Δ*G*_3_). Therefore, Co and Fe sites in CoOOH matrix exhibit different catalytic behaviors for OER. The ΔG_*i*_ of FeCoOOH–V_O_–Co_1_ and FeCoOOH–V_Co_–Co_1_ deviate from the general principle probably due to that they are adjacent to the vacancies and their electronic states are greatly altered.

Scaling relations exist between energetics of OH* and OOH* over the above considered metal sites^36–38^, which are expressed as Δ*G*_OOH*_ = Δ*G*_OH*_ + 2.90 eV (Gibbs free energies, Fig. 4c), Δ*E*_OOH*_ = Δ*E*_OH*_ + 2.87 eV (adsorption energies, Supplementary Fig. 15a), and Δ*E*_OOH_ = Δ*E*_OH_ + 1.03 eV (binding energies, Supplementary Fig. 15b) (Supplementary Table 2). Based on the scaling relation, a universal volcano relationship can be constructed by plotting *η*^OER^ as a function of Δ*G*_O*_ − Δ*G*_OH*_^38^. As shown in Fig. 4d, Fe sites are distributed around the left leg of the volcano which is restricted by the transformation of O* to OOH*, and Co sites are located on the right leg that is determined by the transformation of OH* to O*. Both Fe and Co could serve as OER active centers by analyzing their *η*^OER^, which have been confirmed by previous studies^26,39–41^. Among all considered metal sites in different models, however, the most active center is the Co_2_ site that is adjacent to the vacancy-nearest surface Fe site in FeCoOOH–V_Co_. By comparing the Co_2_ of CoOOH, FeCoOOH, CoOOH–V_Co_ and FeCoOOH–V_Co_, we can see that Fe doping and Co vacancy individually decrease the overpotential from 549 to 380 and 360 mV, respectively, while they jointly push Co_2_ to the top of the volcano, leading to the lowest overpotential of 218 mV among all investigated metal sites. Therefore, the theoretical simulations successfully identify the most active catalytic sites and verify the synergistic effect between Fe dopants and Co vacancies.

To deeply understand how Fe doping and Co vacancy modulate the electronic states of Co_2_ and consequently tune the intermediate binding energy, the partial density of states of Co_2_ 3d in CoOOH, CoOOH–V_Co_, FeCoOOH, FeCoOOH–V_O_, and FeCoOOH–V_Co_ were calculated. As shown in Fig. 4e, an intense negative peak near the Fermi level was observed in CoOOH. Fe doping causes a small shift of the peak away from the Fermi level and Co vacancy induces the splitting of the peak, both of which lead to slightly decreased binding energy of OH*^42^. Remarkably, the combination of Fe doping and Co vacancy greatly decreases the peak intensity near the Fermi level, and therefore results in dramatically weakened binding with OH*. It is important to note that, the low overpotential is achieved due to that Fe doping and Co vacancy jointly have little impact on the binding energy of O* (dashed ellipse in Fig. 4e). In comparison, even though the combination of Fe doping and O vacancy decreases the binding energy of OH* as well, they have a more obvious influence on the binding energy of O*, which leads to increased overpotential and declined catalytic activity.

A contour plot of 3D overpotential surface as a function of Δ*E*_OOH_ − Δ*E*_O_ and Δ*E*_OH_ can be constructed based on the calculated activities of different metal sites. As shown in Fig. 4f, the overpotential decreases along the direction: blue→cyan→green→yellow→red. CoOOH–Co_2_ with a moderate overpotential locates in the green region, and the incorporation of Fe doping and Co vacancy individually moves the point to the green–yellow boundary. FeCoOOH–V_Co_–Co_2_, sitting close to the center of the red region, possesses near-optimal intermediate binding energies, and near-minimum catalytic overpotential that different systems can research in this study. Therefore, Fe doping and Co vacancy work synergistically to approach the activity limit of Co-based catalysts. This contour map provides valuable guidance for the design of efficient OER catalysts via modulating the electronic structures and intermediate binding energies.

In conclusion, different defect structures were incorporated into atomically thin CoSe_2_ nanobelts for OER catalysis. We found that the combination of Fe doping and Co vacancy synergistically optimize the electronic states and intermediate binding energies at Co_2_ site, which is responsible for the dramatically enhanced catalytic activity. This electronic-structure modulation strategy with proper combination of two or more defect structures could efficiently unlock the catalytic power, showing great promise for the rational design of advanced catalysts for various electrochemical reactions.

## Methods

### Material synthesis

Atomically thin CoSe_2_ nanobelts were synthesized via a lamellar intermediate-assisted exfoliation approach. In detail, 14.0 ml DETA was slowly poured into 7.0 ml H_2_O under stirring as severe exothermic reaction occurs. Then, 0.125 g Co(Ac)_2_·4H_2_O and 0.130 g Na_2_SeO_3_ were added and stirred until completely dissolved. After that, hydrothermal reaction was performed at 160 °C for 10 h, and CoSe_2_/DETA lamellar intermediates were finally obtained after reaction. Fe doping was performed by adding 1.0 ml of 0.025 mol L^−1^ Fe(NO_3_)_3_·9H_2_O aqueous solution into the CoSe_2_/DETA intermediates solution, and atomically thin nanobelts were exfoliated from the intermediates in ethanol by using an ultrasonic homogenizer (on time: 2 s, off time: 1 s, output power: 12%, process time: 5 h). Se vacancies were created on the exfoliated Fe-doped CoSe_2_ nanobelts via Ar plasma engraving with a irradiation time of 5 min, a power of 100 W, and a gas flow rate of 50 sccm. Co vacancies were incorporated during the exfoliation of Fe-doped CoSe_2_/DETA intermediates under high-power ultrasonication (on time: 2 s, off time: 1 s, output power: 30%, process time: 2 h). The resulted dispersions were centrifuged at 1000 rmp for 10 min to remove the unexfoliated powders.

### Characterizations

The morphology of nanobelts was observed via STEM (JEOL JEM-ARM200F), and the composition elements were identified by atomic-resolution elemental maps via EDS (Oxford XMax100TLE EDS spectrometer). The element contents before catalysis and after CA test at *η* = 370 mV for 1.5 h were determined by an Optima 2000 DV ICP-emission spectrometer. SAED, XRD (MMA, GBC Scientific Equipment LLC, Hampshire, IL, USA) and Raman scattering (Renishaw 100, 632.8 nm He–Ne laser) were employed to determine the phase structure. The missing lattice atoms were observed by high-resolution HAADF-STEM and analyzed via intensity profile. The electronic states of Co and Fe before catalysis were studied by XPS (PHOIBOS 100 Analyzer from SPECS, Berlin, Germany; Al K_*α*_ X-rays), and those after CA test at *η* = 370 mV for 1.5 h were investigated by EELS (Gatan GIF Quantum ER EELS spectrometer). The EELS characterization was performed at liquid nitrogen temperature to avoid sample damaging and electronic-state changes, and the spectrometer was calibrated against the zero-loss peak at the start of each session. The deconvolution and curve-fitting of the spectra were performed by employing nonlinear least squares fitting tools written in DigitalMicrograph software. The coordination environments of Co and Fe before and after catalysis (CA test at *η* = 320 mV for 0.5 h) were also studied by XANES (National Synchrotron Radiation Laboratory (NSRL), Hefei, China). The Co and Fe L-edges XANES spectra of catalysts were measured at the photoemission end-station at beamline BL10B in the NSRL, Hefei, China. A bending magnet is connected to the beamline, which is equipped with three gratings covering photon energies from 100 to 1000 eV. The samples were kept in the total-electron-yield mode under an ultrahigh vacuum at 5 × 10^−10^ mbar. The resolving power of the grating was typically *E*/Δ*E* = 1000, and the photon flux was 1 × 10^10^ photons per s. Spectra were collected at energies from 770 to 814 eV for Co and 695 to 740 eV for Fe in 0.2 eV energy steps. The XANES raw data were normalized by a procedure consisting of several steps.

### Electrochemical measurements

The catalytic performances were evaluated via the RDE method with continuous rotation at 1200 rpm (CHI 760, Shanghai Chenhua Instruments Co. Ltd.), where O_2_-saturated 1 M NaOH, graphite rod and Hg/HgO electrode were utilized as the electrolyte, counter electrode and reference electrode, respectively. The working electrode was prepared by placing 12 μl of catalyst suspension onto glassy carbon electrode which was dried in a fume cupboard. The catalyst ink was prepared by suspending 2 mg catalyst in 1 ml water/isopropanol/Nafion® mixed solution (3:1:0.2, v/v) under ultrasonication. Before catalytic evaluation, the electrolyte was fully purified with the working electrode via CV cycles between 0.30 and 0.75 V vs. Hg/HgO at 10 mV s^−1^ until a stable response was achieved. New working electrodes were then activated by six CV cycles, during which CoSe_2_ was fully converted into CoOOH. LSV was then recorded within the voltage range of 0.30–1.00 V vs. Hg/HgO at 10 mV s^−1^, and the polarization curves were corrected with 95% *iR*-compensation and then normalized by electrochemical double-layer capacitance that was estimated from the slope of the difference between anodic and cathodic current densities at 0.35 V vs. Hg/HgO against the scan rate (10, 20, 30, 40, 50, and 60 mV s^−1^). After that, the electrodes were stabilized at 0.70 V vs. Hg/HgO for 3 min, and then electrochemical impedance spectroscopy was performed at the same potential with an amplitude of 10 mV and a frequency range of 200 kHz–100 mHz. The catalytic durability was finally assessed by CA test at stepwise potentials of 0.70, 0.75, 0.80, and 0.85 V vs. Hg/HgO for 6 h, and CP test at a constant current density of 10 mA cm^−2^ for 8 h.

Overpotentials were determined by *η*^OER^ = *E*_Hg/HgO_ + (0.098 + 0.059 pH − 1.23) V, where *E*_Hg/HgO_ is the recorded potential vs. Hg/HgO. TOF values were calculated based on TOF = *jS*/4*Fn*, where *j* (A cm^−2^) is the current density at *η* = 280 mV, *S* (cm^2^) is the surface area of glassy carbon electrode, *F* is the Faraday constant (96,485 C mol^−1^), and *n* is the number of moles of the cations assuming all of them are catalytically active. Tafel plots were derived from the polarization curves in low overpotential regions by plotting overpotential against log(current density). The error bars of reported overpotentials, current densities and TOFs were obtained from five replicates after discarding the maximum and minimum values.

### Computational methods

In this work, the crystal structure of CoOOH was chosen to build up the periodical surfaces including Fe doping and Co/O vacancy models. The high-index (01-12) facet was adopted as the surface termination^36,37^, as it exhibits theoretical activities in good agreement with the experimental results. The periodic surface was 14.79 Ǻ × 15.22 Ǻ with a vacuum slab of 18 Å in thickness to separate the layer from its periodic images. All the calculations were performed by spin-polarized plane-wave DFT, as implemented in the CASTEP program^43^. The geometry optimizations were performed using the BFGS algorithm. The Perdew–Burke–Ernzerhof exchange-correlation functional within the generalized gradient approximation as well as ultrasoft pseudopotentials was selected^44,45^. The cut-off energy for the plane-wave basis and the Brilliouin zone k-points were chosen as 340.0 eV and 0.04 Ǻ^−1^ spacing in the Monkhorst–Pack scheme^46^, respectively, which causes little difference in the calculation results when further increase. To sufficiently consider the on-site Columbic repulsion between the d electrons, the Hubbard *U* corrections were applied to transition metal d-electrons and the values of *U*–*J* parameters for Co (3.42) and Fe (3.29) atoms were taken from the reference^47^. The convergence criteria for the total energy, forces, stress, atomic displacement, and self-consistent field iterations were set to 1 × 10^−5^ eV atom^−1^, 3 × 10^−2^ eV Å^−1^, 5 × 10^−2^ GPa, 1 × 10^−3^ Å, and 1 × 10^−6^ eV atom^−1^, respectively.

The OER reaction steps and Gibbs free energy changes can be expressed by^37^

1 $$\begin{array}{l}{\mathrm{H}}_{\mathrm{2}}{\mathrm{O + }} \ast \to {\mathrm{OH}} \ast {\mathrm{ + e}}^{\mathrm{ - }}{\mathrm{ + H}}^{\mathrm{ + }}\\ \Delta {G}_{\mathrm{1}} = \Delta {G}_{{\mathrm{OH}} \ast }{ - eU + }{\it{k}}_{\mathrm{B}}{{T{\mathrm{ln10}} \times \mathrm{pH}}}\end{array},$$

2 $$\begin{array}{l}{\mathrm{OH}} \ast \to {\mathrm{O}} \ast + {\mathrm{e}}^ - + {\mathrm{H}}^ + \\ \Delta {G}_2 = \Delta {G}_{{\mathrm{O}} \ast } - \Delta {G}_{{\mathrm{OH}} \ast } - {eU} + k_{\mathrm{B}}{T{\mathrm{ln10}}} \times {\mathrm{pH}}\end{array},$$

3 $$\begin{array}{l}{\mathrm{O}} \ast + {\mathrm{H}}_2{\mathrm{O}} \to {\mathrm{OOH}} \ast + {\mathrm{e}}^ - + {\mathrm{H}}^ + \\ \Delta {G}_3 = \Delta {G}_{{\mathrm{OOH}} \ast } - \Delta {G}_{{\mathrm{O}} \ast } - {eU} + k_{\mathrm{B}}{T\mathrm{ln}}10 \times {\mathrm{pH}}\end{array},$$

4 $$\begin{array}{l}{\mathrm{OOH}} \ast \to \ast + {\mathrm{O}}_2 + {\mathrm{e}}^ - + {\mathrm{H}}^ + \\ \Delta {G}_4 = 4.92\,{\mathrm{eV}} - \Delta {G}_{{\mathrm{OOH}} \ast } - eU + k_{\mathrm{B}}{T\mathrm{ln10}} \times {\mathrm{pH}}\end{array},$$

where * represents an active site on (01-12) facet, and ΔG_*i*_ (*i* = OH*, O*, and OOH*) are Gibbs free energies of OER intermediates. The theoretical overpotential under standard conditions (*T* = 298.15 K, *p* = 1 bar, pH = 0) is then given by^37^

5 $${\upeta}^{{\mathrm{OER}}} = {\mathrm{max}}\left[ {\Delta {G}_1,\,\Delta {G}_2,\,\Delta {G}_3,\,\Delta {G}_4} \right]/e - 1.23\,{\mathrm{V}}.$$

The Gibbs free energies, ΔG_*i*_, were determined by the adsorption energies combined with corrections for zero-point energy and entropy, according to Δ*G*_*i*_ = Δ*E*_*i*_ + ΔZPE_*i*_ − *T*Δ*S*_*i*_. The ZPE and TS were calculated using DFT calculations of vibrational frequencies and using standard tables for gas-phase molecules^48^. The ZPEs for H_2_, H_2_O, *OH, *O, and *OOH are 0.27, 0.56, 0.35, 0.05, and 0.41 eV, respectively, and the TS corrections for H_2_ and H_2_O are 0.41 and 0.67 eV, respectively (we assume *S* = 0 for the adsorbates on coordinatively unsaturated sites). The adsorption energies of OER intermediates, Δ*E*_*i*_ (*i* = OH*, O*, and OOH*), were calculated relative to H_2_O and H_2_ (at *U* = 0 and pH = 0)

6 $$\Delta {E}_{{\mathrm{OH}} \ast } = {E}_{{\mathrm{OH}} \ast } - {E}_ \ast - \left( {{E}_{{\mathrm{H2O}}} - 1/2{E}_{{\mathrm{H}}2}} \right),$$

7 $$\Delta {E}_{{\mathrm{O}} \ast } = {E}_{{\mathrm{O}} \ast } - {E}_ \ast - \left( {{E}_{{\mathrm{H2O}}} - {E}_{{\mathrm{H2}}}} \right),$$

8 $$\Delta {E}_{{\mathrm{OOH}} \ast } = {E}_{{\mathrm{OOH}} \ast } - {E}_ \ast - \left( {2E_{{\mathrm{H2O}}} - 3/2E_{{\mathrm{H2}}}} \right).$$

The binding energies, Δ*E*_*j*_ (*j* = OH, O, and OOH), were calculated directly by Δ*E*_*j*_ = *E*_j*_ − *E*_*j*_ − E_*_,

where * represents an active site on (01-12) facet, and *E* is the total energy calculated by using the spin polarization DFT method.

## Supplementary information

Supplementary Info

## Data Availability

The data that support the findings of this study are available from the corresponding author upon reasonable request.

## References

[1] Suen NT (2017). Electrocatalysis for the oxygen evolution reaction: recent development and future perspectives. Chem. Soc. Rev..

[2] Wu T (2019). Iron-facilitated dynamic active-site generation on spinel CoAl_2_O_4_ with self-termination of surface reconstruction for water oxidation. Nat. Catal..

[3] Jin H (2018). Emerging two-dimensional nanomaterials for electrocatalysis. Chem. Rev..

[4] Pan X, Yang MQ, Fu X, Zhang N, Xu YJ (2013). Defective TiO_2_ with oxygen vacancies: synthesis, properties and photocatalytic applications. Nanoscale.

[5] Yan J (2019). Single atom tungsten doped ultrathin α-Ni(OH)_2_ for enhanced electrocatalytic water oxidation. Nat. Commun..

[6] Peng S (2018). Necklace-like multishelled hollow spinel oxides with oxygen vacancies for efficient water electrolysis. J. Am. Chem. Soc..

[7] Li H (2015). Fe-doped CoSe_2_ nanoparticles encapsulated in N-doped bamboo-like carbon nanotubes as an efficient electrocatalyst for oxygen evolution reaction. Electrochim. Acta.

[8] Zhang J-Y (2017). Rational design of cobalt-iron selenides for highly efficient electrochemical water oxidation. ACS Appl. Mater. Interfaces.

[9] Gao MR, Yao WT, Yao HB, Yu SH (2009). Synthesis of unique ultrathin lamellar mesostructured CoSe_2_-amine (protonated) nanobelts in a binary solution. J. Am. Chem. Soc..

[10] Stevens MB (2017). Reactive Fe-sites in Ni/Fe (oxy)hydroxide are responsible for exceptional oxygen electrocatalysis activity. J. Am. Chem. Soc..

[11] Chang A, Zhang C, Yu Y, Yu Y, Zhang B (2018). Plasma-assisted synthesis of NiSe_2_ ultrathin porous nanosheets with selenium vacancies for supercapacitor. ACS Appl. Mater. Interfaces.

[12] Liu Y (2014). Low overpotential in vacancy-rich ultrathin CoSe_2_ nanosheets for water oxidation. J. Am. Chem. Soc..

[13] Zheng YR (2018). Doping-induced structural phase transition in cobalt diselenide enables enhanced hydrogen evolution catalysis. Nat. Commun..

[14] Gui Y (2019). Manipulating the assembled structure of atomically thin CoSe_2_ nanomaterials for enhanced water oxidation catalysis. Nano Energy.

[15] Dou Y (2016). Atomic layer-by-layer Co_3_O_4_/graphene composite for high performance lithium-ion batteries. Adv. Energy Mater..

[16] Lesnyak V, Brescia R, Messina GC, Manna L (2015). Cu vacancies boost cation exchange reactions in copper selenide nanocrystals. J. Am. Chem. Soc..

[17] Zhou Q (2018). Active-site-enriched iron-doped nickel/cobalt hydroxide nanosheets for enhanced oxygen evolution reaction. ACS Catal..

[18] Zheng YR (2015). An efficient CeO_2_/CoSe_2_ nanobelt composite for electrochemical water oxidation. Small.

[19] Zhou Y (2017). Co_3_O_4_@(Fe-doped)Co(OH)_2_ microfibers: facile synthesis, oriented-assembly, formation mechanism, and high electrocatalytic activity. ACS Appl. Mater. Interfaces.

[20] Zhao X (2017). Engineering the electrical conductivity of lamellar silver-doped cobalt(II) selenide nanobelts for enhanced oxygen evolution. Angew. Chem. Int. Ed..

[21] Jin H (2017). Fe incorporated α-Co(OH)_2_ nanosheets with remarkably improved activity towards the oxygen evolution reaction. J. Mater. Chem. A.

[22] Li J (2018). Fe-doped CoSe_2_ nanoparticles encapsulated in N-doped bamboo-like carbon nanotubes as an efficient electrocatalyst for oxygen evolution reaction. Electrochim. Acta.

[23] Liang L (2015). Metallic single-unit-cell orthorhombic cobalt diselenide atomic layers: robust water-electrolysis catalysts. Angew. Chem. Int. Ed..

[24] Gao MR (2014). CoSe_2_ nanobelts composite catalyst for efficient water oxidation. ACS Nano.

[25] Xu H (2018). Phosphorus-doped cobalt-iron oxyhydroxide with untrafine nanosheet structure enable efficient oxygen evolution electrocatalysis. J. Colloid Interface Sci..

[26] Zhang L (2017). Ultrathin iron-cobalt oxide nanosheets with abundant oxygen vacancies for the oxygen evolution reaction. Adv. Mater..

[27] Xiao C, Lu X, Zhao C (2014). Unusual synergistic effects upon incorporation of Fe and/or Ni into mesoporous Co_3_O_4_ for enhanced oxygen evolution. Chem. Commun..

[28] Gao MR, Xu YF, Jiang J, Zheng YR, Yu SH (2012). Water oxidation electrocatalyzed by an efficient Mn_3_O_4_/CoSe_2_ nanocomposite. J. Am. Chem. Soc..

[29] Li N (2017). Influence of iron doping on tetravalent nickel content in catalytic oxygen evolving films. Proc. Natl Acad. Sci. USA.

[30] Jiang H, He Q, Zhang Y, Song L (2018). Structural self-reconstruction of catalysts in electrocatalysis. Acc. Chem. Res..

[31] Guo C (2017). Engineering high-energy interfacial structures for high-performance oxygen-involving electrocatalysis. Angew. Chem. Int. Ed..

[32] Zhang S (2010). Determination of manganese valence states in (Mn^3+^, Mn^4+^) minerals by electron energy-loss spectroscopy. Am. Mineral..

[33] Ling T (2017). Activating cobalt(II) oxide nanorods for efficient electrocatalysis by strain engineering. Nat. Commun..

[34] Cheng J (2018). Enhanced insulating behavior in the Ir-vacant Sr_2_Ir_1-x_O_4_ system dominated by the local structure distortion. J. Synchrotron Rad..

[35] Cao L (2019). Identification of single-atom active sites in carbon-based cobalt catalysts during electrocatalytic hydrogen evolution. Nat. Catal..

[36] Bajdich M, García-Mota M, Vojvodic A, Nørskov JK, Bell AT (2013). Theoretical investigation of the activity of cobalt oxides for the electrochemical oxidation of water. J. Am. Chem. Soc..

[37] Friebel D (2015). Identification of highly active Fe sites in (Ni,Fe)OOH for electrocatalytic water splitting. J. Am. Chem. Soc..

[38] Man IC (2011). Universality in oxygen evolution electrocatalysis on oxide surfaces. ChemCatChem.

[39] Burke MS (2015). Cobalt-iron (oxy)hydroxide oxygen evolution electrocatalysts: the role of structure and composition on activity, stability, and mechanism. J. Am. Chem. Soc..

[40] Smith RDL (2017). Spectroscopic identification of active sites for the oxygen evolution reaction on iron-cobalt oxides. Nat. Commun..

[41] Zhang B (2016). Homogeneously dispersed multimetal oxygen-evolving catalysts. Science.

[42] Qiu B (2018). Fabrication of nickel-cobalt bimetal phosphide nanocages for enhanced oxygen evolution catalysis. Adv. Funct. Mater..

[43] Clark SJ (2005). First principles methods using CASTEP. Z. Kristallogr..

[44] Vanderbilt D (1990). Soft self-consistent pseudopotentials in a generalized eigenvalue formalism. Phys. Rev. B.

[45] Perdew JP, Burke K, Ernzerhof M (1996). Generalized gradient approximation made simple. Phys. Rev. Lett..

[46] Monkhorst HJ, Park JD (1976). Special points for Brillouin-zone intergrations. Phys. Rev. B.

[47] Xu H, Cheng D, Cao D, Zeng XC (2018). A universal principle for a rational design of single-atom electrocatalysts. Nat. Catal..

[48] Valdés Á, Qu ZW, Kroes GJ, Rossmeisl J, Nørskov JK (2008). Oxidation and photo-oxidation of water on TiO_2_ surface. J. Phys. Chem. C.

