# Supplementary material for: Approaching the activity limit of CoSe2 for oxygen evolution via Fe doping and Co vacancy
Source: Nat Commun. 2020 Apr 3;11:1664. doi: 10.1038/s41467-020-15498-0 (PMC7125230; doi:10.1038/s41467-020-15498-0)
Supplement: Supplementary file 1 — Supplementary Info [file 41467_2020_15498_MOESM1_ESM.pdf]

Supplementary Information

Approaching the Activity Limit of $CoSe_2$ for Oxygen Evolution via Fe Doping and Co Vacancy

Dou et al.

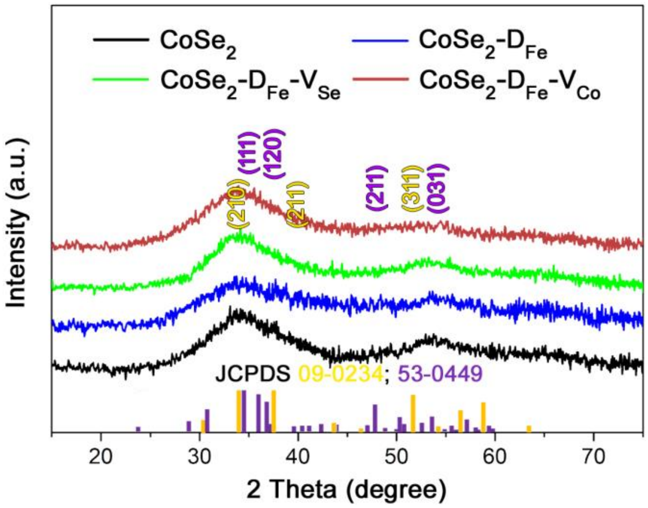

Supplementary Fig. 1 | X-ray diffraction patterns of different catalysts showing the mixed cubic (JCPDS 09-0234) and orthorhombic (JCPDS 53-0449) phases of $CoSe_2$.

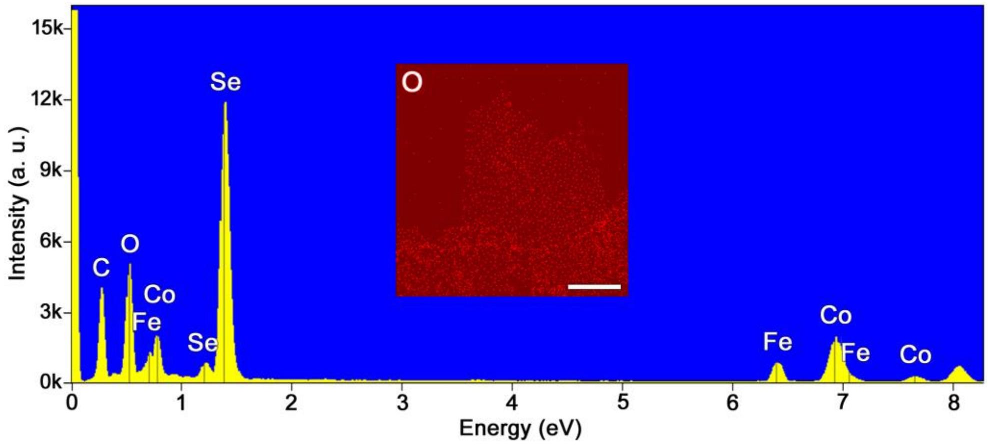

Supplementary Fig. 2 | Energy dispersive X-ray spectroscopy pattern and elemental mapping showing the existence of O in $CoSe_2$-D_{Fe}-V_{Co}. Scale bar, 300 nm.

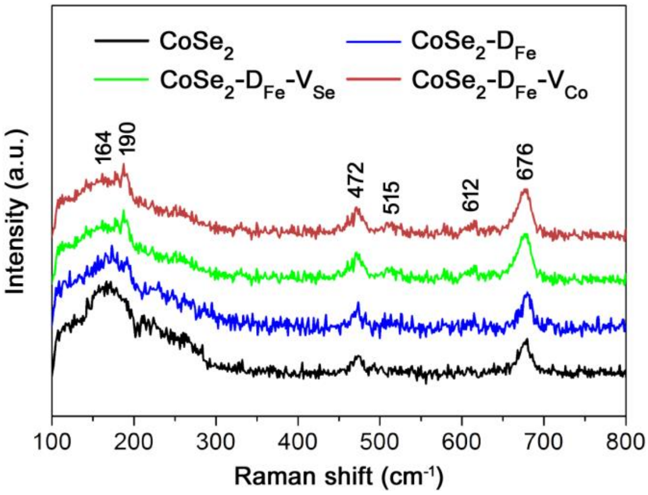

Supplementary Fig. 3 | Raman spectra of $CoSe_2$ matrix with oxide impurities.

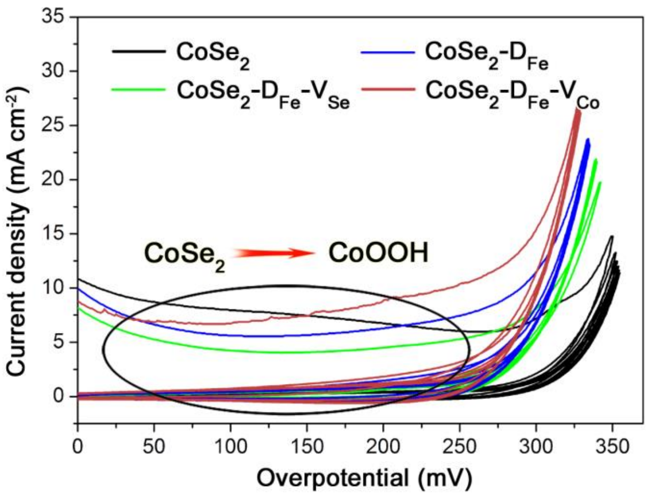

Supplementary Fig. 4 | Cyclic voltammetry activation of different catalysts. The catalysts undergo phase conversion in alkaline solution at anodic potentials.

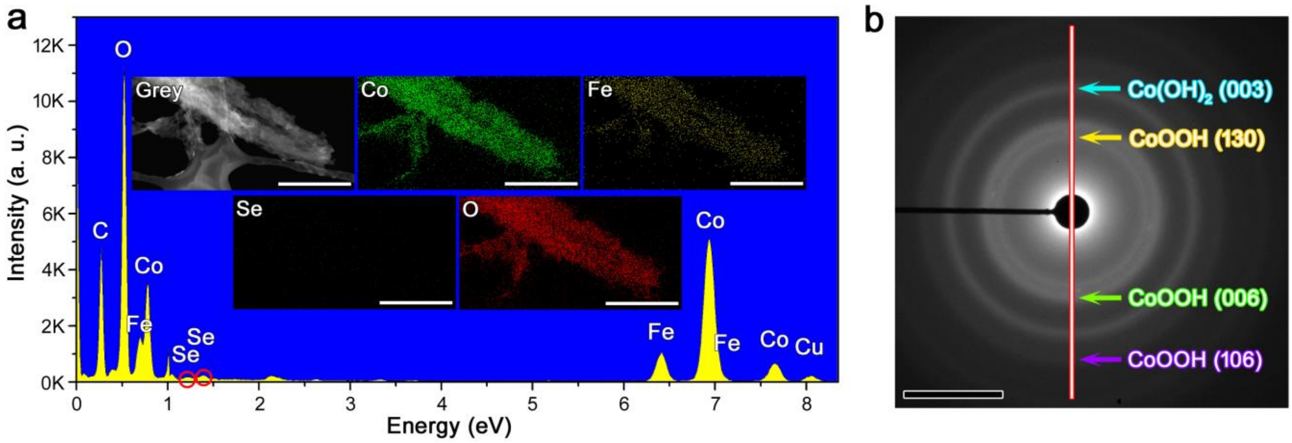

Supplementary Fig. 5 | Phase characterization of $CoSe_2$-D_{Fe}-V_{Co} after cyclic voltammetry activation. **a** Energy dispersive X-ray spectroscopy pattern and elemental mapping showing that almost no Se can be detected. Scale bar, 300 nm. **b** Selected area electron diffraction pattern illustrating the formation of CoOOH. Scale bar, 5 1/nm.

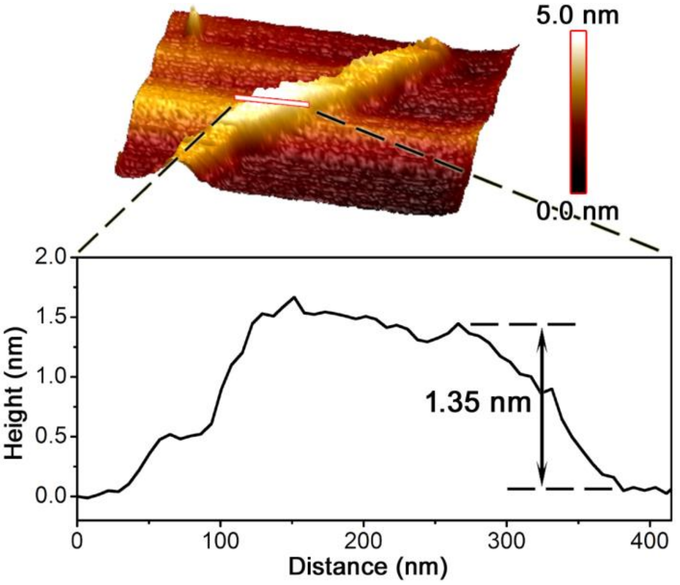

Supplementary Fig. 6 | Atomic force microscopy image of $CoSe_2$-D_{Fe}-V_{Co} after cyclic voltammetry activation and height profile along the white line in the image.

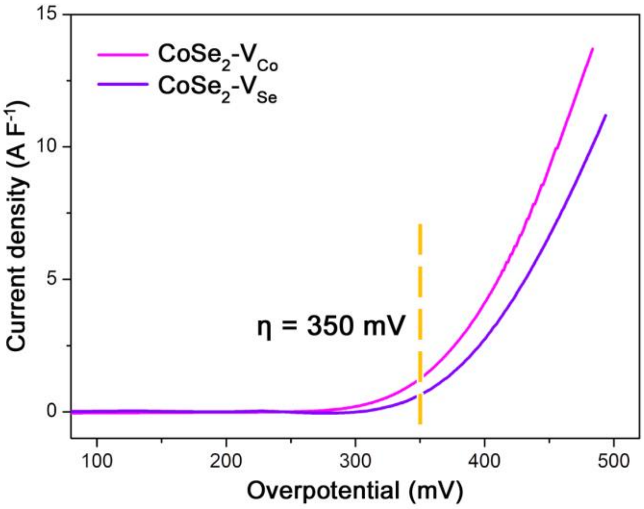

Supplementary Fig. 7 | Linear sweep voltammetry curves of $CoSe_2$-V_{Co} and $CoSe_2$-V_{Se}.

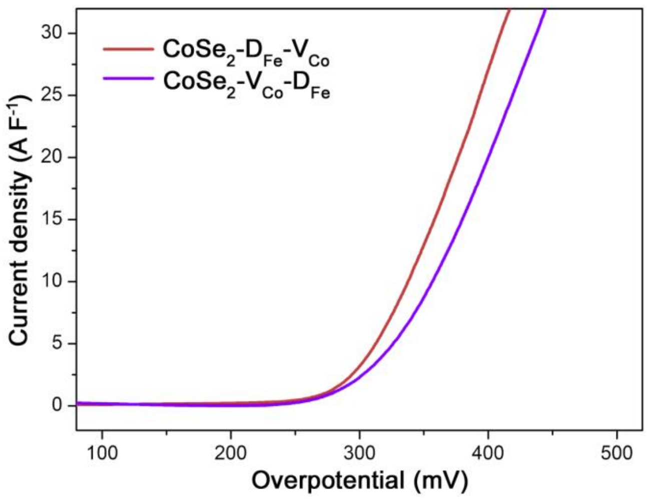

Supplementary Fig. 8 | Performance comparison by changing the sequence of Fe doping and Co vacancy.

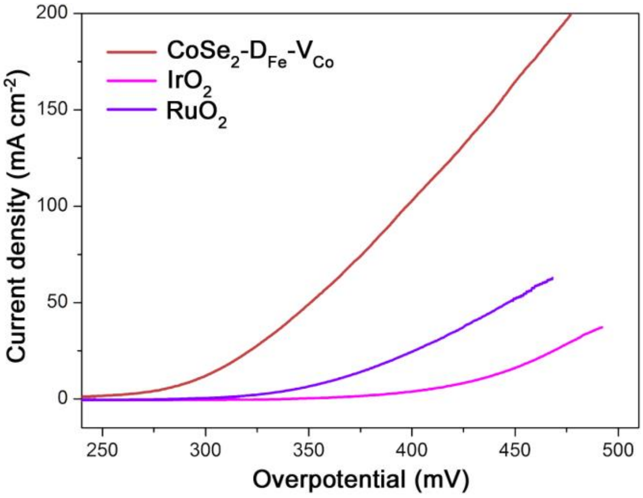

Supplementary Fig. 9 | Linear sweep voltammetry curves of $CoSe_2$-D_{Fe}-V_{Co}, IrO_2 and RuO_2.

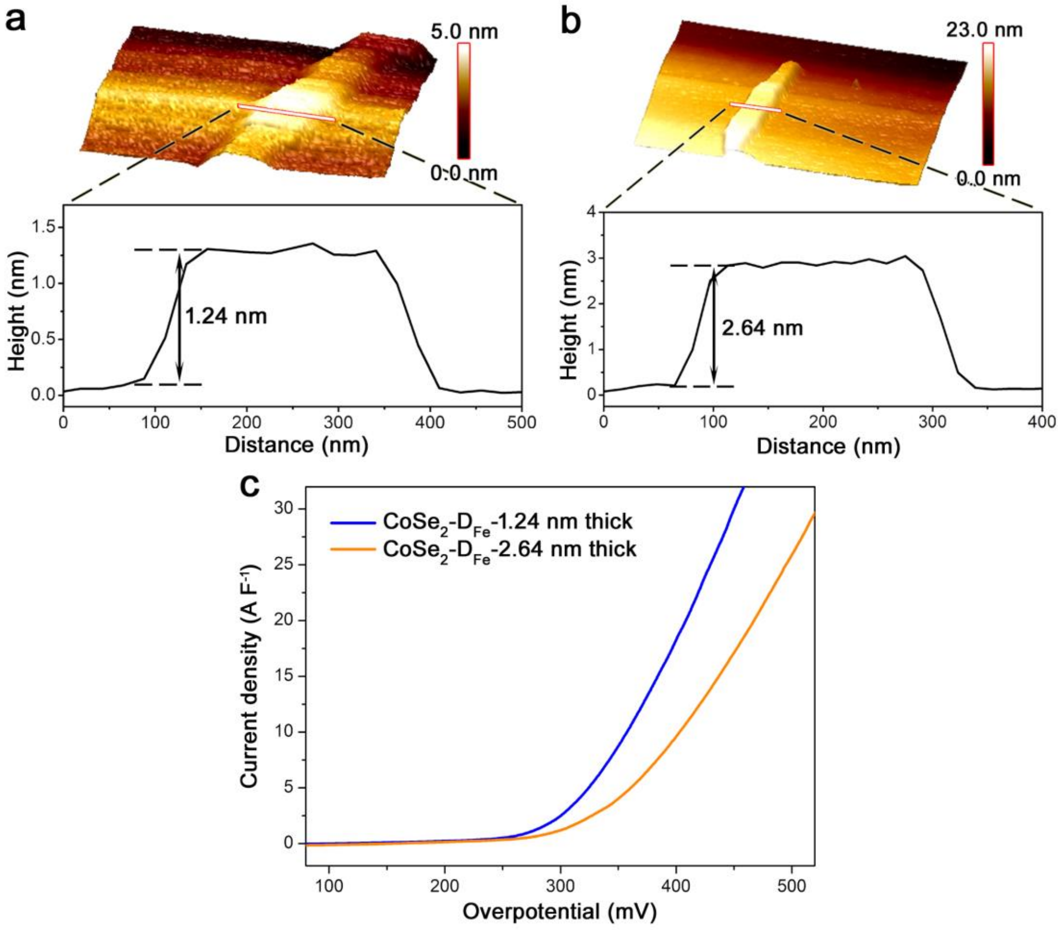

Supplementary Fig. 10 | Effect of nanobelt thickness on catalytic performance for oxygen evolution reaction. a, **b** Atomic force microscopy images and height profiles of $CoSe_2$-D_{Fe}-1.24 nm and $CoSe_2$-D_{Fe}-2.64 nm, prepared by low-power ultrasonication for 5 h and 2 h, respectively. **c** Linear sweep voltammetry curves of $CoSe_2$-D_{Fe}-1.24 nm and $CoSe_2$-D_{Fe}-2.64 nm normalized by electrochemical double-layer capacitance.

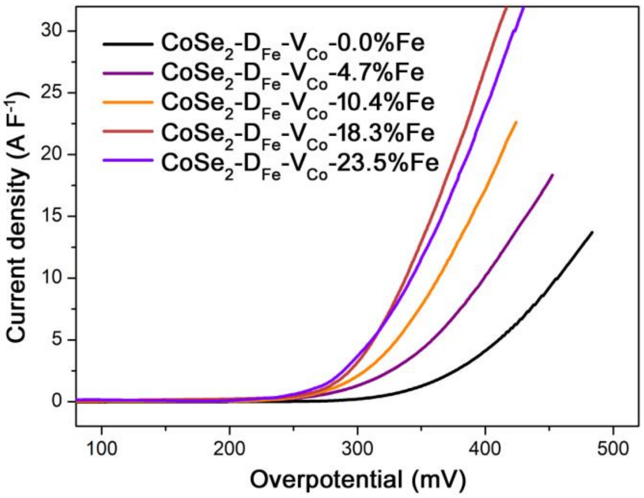

Supplementary Fig. 11 | Effect of Fe doping level on OER catalytic activity. The $CoSe_2$-D_{Fe}-V_{Co} catalysts with different Fe doping levels (0.0%, 4.7%, 10.4%, 18.3% and 23.5%) were fabricated by adding respectively 0.00, 0.25, 0.50, 1.00 and 2.00 ml of 0.025 mol L^{-1} $Fe(NO_3)_3$ aqueous solution into the $CoSe_2$/diethylenetriamine intermediates solution after hydrothermal reaction.

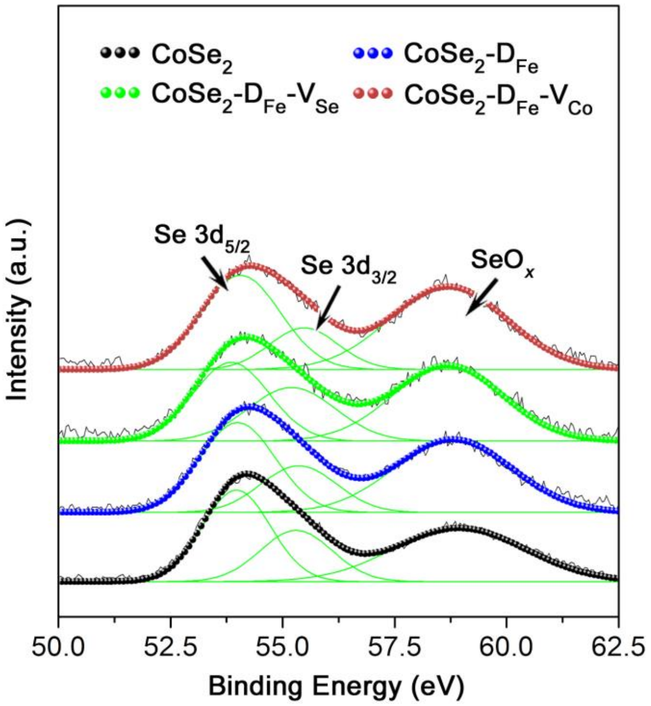

Supplementary Fig. 12 | Se 3d X-ray photoelectron spectroscopy spectra of primary catalysts.

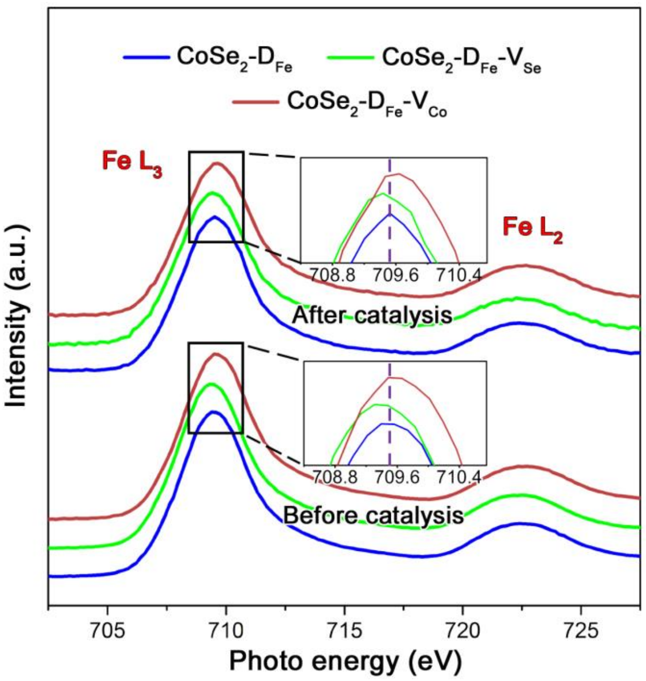

Supplementary Fig. 13 | Fe L-edge X-ray absorption near-edge structure spectra before and after catalysis showing the energy shift caused by defects.

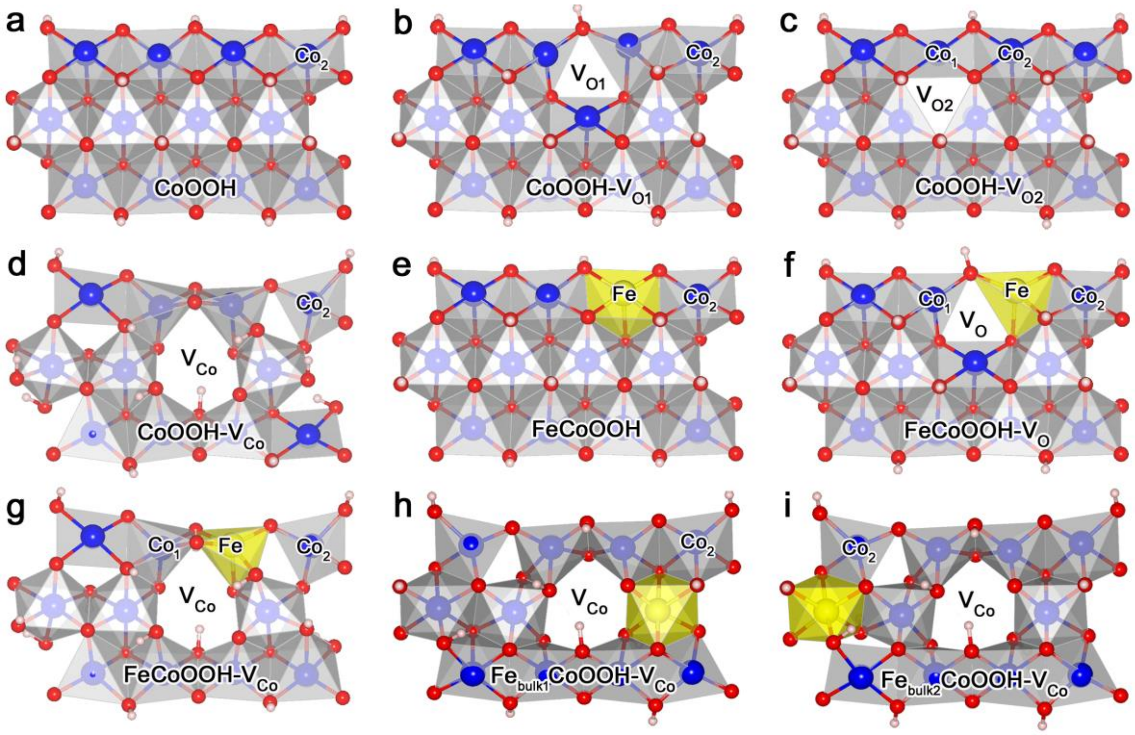

Supplementary Fig. 14 | Potential active sites on (01-12) facets considered by Hubbard-corrected density functional theory calculations. **a** Co_2 site in CoOOH. **b** Co_2 site in CoOOH-V_{O1}. **c** Co_1 and Co_2 sites in CoOOH-V_{O2}. **d** Co_2 site in CoOOH-V_{Co}. **e** Fe and Co_2 sites in FeCoOOH. **f** Fe, Co_1 and Co_2 sites in FeCoOOH-V_O. **g** Fe, Co_1 and Co_2 sites in FeCoOOH-V_{Co}. **h** Co_2 site in Fe_{bulk1}CoOOH-V_{Co}. **i** Co_2 site in Fe_{bulk2}-CoOOH-V_{Co}.

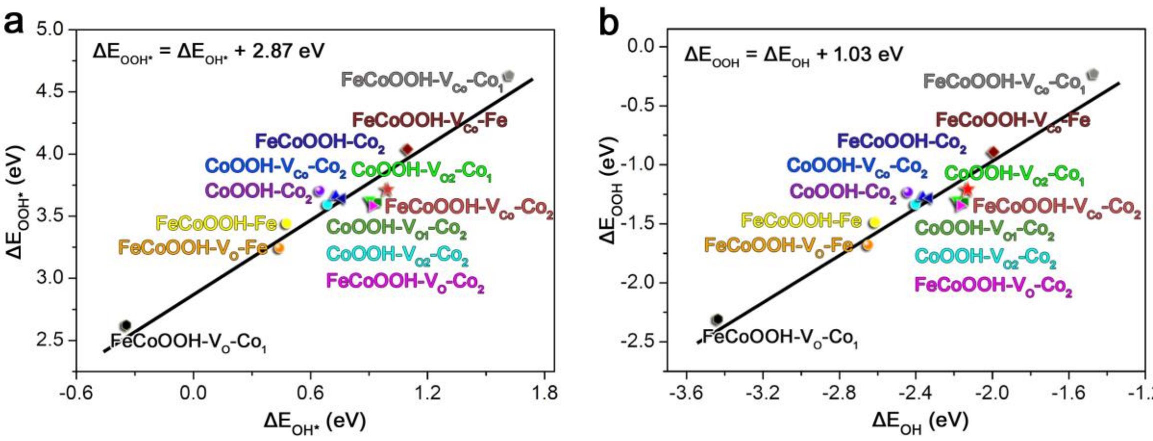

Supplementary Fig. 15 | Scaling relations between energetics of OH* and OOH* intermediates.

a Adsorption energy relation expressed by $\Delta E_{OOH*} = \Delta E_{OH*} + 2.87$ eV. **b** Binding energy relation expressed by $\Delta E_{OOH} = \Delta E_{OH} + 1.03$ eV.

Supplementary Table 1 | Elemental contents before and after catalysis determined by inductively coupled plasma atomic emission spectroscopy.

Atom%		Co	Fe	Se
**$CoSe_2$**	Before	42.5	-	57.5
	After	100.0	-	-
**$CoSe_2$-D_{Fe}**	Before	35.2	7.9	56.9
	After	81.6	18.4	-
**$CoSe_2$-D_{Fe}-V_{Se}**	Before	35.7	8.1	56.2
	After	81.5	18.5	-
**$CoSe_2$-D_{Fe}-V_{Co}**	Before	34.5	7.7	57.8
	After	81.7	18.3	-

Supplementary Table 2 | Calculated energetics and overpotentials of different metal sites.

	Binding energies (eV)			Adsorption energies (eV)		
	ΔE_{OH}	ΔE_{O}	ΔE_{OOH}	ΔE_{OH*}	ΔE_{O*}	ΔE_{OOH*}
**CoOOH-Co$_2$**	-2.4420	-2.8235	-1.2348	0.6490	2.7990	3.6957
**CoOOH-V$_{O1}$-Co$_2$**	-2.1492	-2.4708	-1.3214	0.9420	3.1510	3.6091
**CoOOH-V$_{O2}$-Co$_2$**	-2.4048	-2.6862	-1.3384	0.6870	2.9360	3.5921
**CoOOH-V$_{O2}$-Co$_1$**	-2.1881	-2.5645	-1.3035	0.9030	3.0580	3.6270
**CoOOH-V$_{Co}$-Co$_2$**	-2.3627	-2.9336	-1.2681	0.7290	2.6880	3.6624
**FeCoOOH-Co$_2$**	-2.3370	-2.8874	-1.2864	0.7550	2.7350	3.6441
**FeCoOOH-Fe**	-2.6123	-3.7023	-1.4920	0.4790	1.9200	3.4385
**FeCoOOH-V$_{O}$-Co$_2$**	-2.1786	-2.5392	-1.3350	0.9130	3.0830	3.5955
**FeCoOOH-V$_{O}$-Co$_1$**	-3.4347	-5.4055	-2.3093	-0.3430	0.2170	2.6212
**FeCoOOH-V$_{O}$-Fe**	-2.6513	-3.6679	-1.6828	0.4400	1.9540	3.2477
**FeCoOOH-V$_{Co}$-Co$_2$**	-2.1335	-2.8095	-1.2132	0.9950	2.8130	3.7173
**FeCoOOH-V$_{Co}$-Co$_1$**	-1.4697	-1.9658	-0.2300	1.6220	3.6560	4.6306
**FeCoOOH-V$_{Co}$-Fe**	-1.9944	-2.9599	-0.8939	1.0970	2.6620	4.0366
**Fe$_{bulk1}$CoOOH-V$_{Co}$-Co$_2$**	-2.6712	-3.4722	-1.9995	0.4203	2.1509	2.9310
**Fe$_{bulk2}$CoOOH-V$_{Co}$-Co$_2$**	-2.7096	-3.4272	-2.0428	0.3819	2.1948	2.8877

Gibbs free energies (eV)				Gibbs free energy changes (eV)				η^{OER}
ΔG_{OH*}	ΔG_{O*}	ΔG_{OOH*}	ΔG_{O2}	ΔG_1	ΔG_2	ΔG_3	ΔG_4	(mV)
1.0392	2.8185	4.1157	4.9200	1.0392	1.7793	1.2972	0.8043	549
1.3323	3.1712	4.0291	4.9200	1.3323	1.8389	0.8579	0.8909	609
1.0767	2.9558	4.0121	4.9200	1.0767	1.8791	1.0563	0.9079	649
1.2934	3.0775	4.0470	4.9200	1.2934	1.7841	0.9695	0.8730	554
1.1188	2.7084	4.0824	4.9200	1.1188	1.5896	1.3740	0.8376	360
1.1445	2.7546	4.0641	4.9200	1.1445	1.6101	1.3095	0.8559	380
0.8692	1.9397	3.8585	4.9200	0.8692	1.0705	1.9188	1.0615	689
1.3029	3.1028	4.0155	4.9200	1.3029	1.7999	0.9127	0.9045	570
0.0468	0.2365	3.0412	4.9200	0.0468	0.1897	2.8047	1.8788	1575
0.8302	1.9741	3.6677	4.9200	0.8302	1.1439	1.6936	1.2523	464
1.3848	2.8325	4.1373	4.9200	1.3848	1.4477	1.3048	0.7827	218
2.0118	3.6762	5.0506	4.9200	2.0118	1.6644	1.3744	-0.1306	782
1.4871	2.6821	4.4566	4.9200	1.4871	1.1950	1.7745	0.4634	545
0.8103	2.1709	3.3510	4.9200	0.8103	1.3606	1.1801	1.5690	339
0.7719	2.2148	3.3077	4.9200	0.7719	1.4429	1.0929	1.6123	382
